# The Other Function: Class II-Restricted Antigen Presentation by B Cells

**DOI:** 10.3389/fimmu.2017.00319

**Published:** 2017-03-23

**Authors:** Lital N. Adler, Wei Jiang, Kartik Bhamidipati, Matthew Millican, Claudia Macaubas, Shu-chen Hung, Elizabeth D. Mellins

**Affiliations:** ^1^Department of Pediatrics, Stanford University, Stanford, CA, USA; ^2^Program in Immunology, Stanford University, Stanford, CA, USA; ^3^Stanford University, Stanford, CA, USA

**Keywords:** B cell, MHC class II, antigen processing, antigen presentation, invariant chain, HLA-DM, HLA-DO, germinal center

## Abstract

Mature B lymphocytes (B cells) recognize antigens using their B cell receptor (BCR) and are activated to become antibody-producing cells. In addition, and integral to the development of a high-affinity antibodies, B cells utilize the specialized major histocompatibility complex class II (MHCII) antigen presentation pathway to process BCR-bound and internalized protein antigens and present selected peptides in complex with MHCII to CD4+ T cells. This interaction influences the fate of both types of lymphocytes and shapes immune outcomes. Specific, effective, and optimally timed antigen presentation by B cells requires well-controlled intracellular machinery, often regulated by the combined effects of several molecular events. Here, we delineate and summarize these events in four steps along the antigen presentation pathway: (1) antigen capture and uptake by B cells; (2) intersection of internalized antigen/BCRs complexes with MHCII in peptide-loading compartments; (3) generation and regulation of MHCII/peptide complexes; and (4) exocytic transport for presentation of MHCII/peptide complexes at the surface of B cells. Finally, we discuss modulation of the MHCII presentation pathway across B cell development and maturation to effector cells, with an emphasis on the shaping of the MHCII/peptide repertoire by two key antigen presentation regulators in B cells: HLA-DM and HLA-DO.

## Introduction

In addition to their role as secretors of antibodies, B cells function as professional antigen-presenting cells (APCs) for CD4+ T cells by expressing cell-surface major histocompatibility complex class II (MHCII) molecules with bound peptide, the ligand of the α/β T cell receptor. MHCII-restricted antigen presentation by B cells plays a role in multiple immunological events. For example, recent evidence supports a model of sequential class II-restricted antigen presentation by thymic B cells to CD4+ thymocytes (1). In this scenario, resting IgD+/IgM+ B cells enter the thymus and undergo cognate interactions with CD4+ thymocytes and CD40-mediated B cell activation. This increases MHCII, CD80, and Aire expression, allowing B cell presentation of Aire-dependent antigens and negative selection of self-antigen reactive thymocytes. In another scenario, B cells contribute to the magnitude of primary CD4+ T cell responses (2) and to the development and expansion of Th2 cytokine-secreting CD4+ T effectors (T_EFF_) (3). B cell division, hypermutation of immunoglobulin genes and engagement of T follicular helper cells (T_FH_) in the germinal center (GC) are directly proportional to the amount of antigen captured and presented on MHCII (4, 5). Antigen presentation by B cells also is critical to the generation and survival of memory CD4+ T cells (6). Recent work revealed a memory checkpoint when CD4+ T_EFF_ require cognate antigen recognition for further differentiation into memory cells (7); at this checkpoint, B cell APC are required for driving T_EFF_ to T_FH_ (8). In murine models of type 1 diabetes and multiple sclerosis, antigen presentation by B cells contributes to disease pathogenesis (9, 10). Related to this, blockade of B cell antigen presentation is implicated as one mechanism underlying efficacy of anti-CD20 therapy in CD4+ T cell-mediated diseases, including autoimmune hepatitis, rheumatoid arthritis, and multiple sclerosis and mouse models of these diseases [(11–14) and references therein]. In addition, Jackson et al. have proposed that, in certain genetic settings, B cell presentation of self-antigens can initiate a break in CD4+ T cell tolerance (15). In summary, MHCII-restricted antigen presentation by B cells is critical to immune function in health and disease, highlighting the importance of a comprehensive understanding of the associated molecular mechanisms.

Here, we review current information on B cell antigen uptake, processing, and presentation as MHCII/peptide complexes at the cell membrane. We discuss antigen encounter in secondary lymphoid organs (SLOs), the role of the B cell receptor (BCR) in capturing antigen, and B cell polarization, in the case of antigen capture from the surface of other cells. We next cover BCR internalization, the targeting of BCR/antigen complexes to subcellular compartments for interaction with MHCII, the roles of B cell proteases in invariant chain (Ii) degradation and generation of the peptide pool for MHCII loading. We review the shaping of the MHCII/peptide repertoire by HLA-DM and HLA-DO and the MHCII/peptide complex transport to the surface of B cells. Finally, the development and modulation of the MHCII presentation pathway across the stages of B cell differentiation are discussed.

In the mouse, B cells can be separated into B-1 and B-2 subsets, and recent studies argue that these are distinct lineages (16). B-1 cells (which likely have a human equivalent functionally) mostly arise in the fetal liver, self-renew in the periphery, and represent a small population that acts to achieve a rapid, early immune responses independent of T cell help (12, 16, 17). B-2 cells (in mice and humans) are mainly produced in the bone marrow throughout life and predominate in the B cell pool of SLO. B-2 cells are a key component of adaptive immune system, exhibiting antigen specificity and memory and mediating both T cell-independent and T cell-dependent antibody production. This review focuses on the class II antigen presentation pathway in B-2 cells (referred to as B cells in what follows).

## The BCR and Antigen Encounter

A key component of immune surveillance is the circulation of naïve, mature B cells through SLOs. About 45% of naïve B cells migrate to the spleen to sample antigens present in the blood; a roughly equal number move to the lymph nodes to sample antigens drained from tissues, and the remainder move mostly to mucosa-associated lymphoid tissues (MALT), a key interface between the host and its environment (18). Within SLO, the B cells localize to B cell follicles in a chemokine (CXCR13)-dependent manner and begin to sample antigen. How antigen traffics to the B cell zone depends on the SLO. In MALT, antigen is passed across the epithelial barrier into B cell zones (19). In the spleen, antigen draining from the splenic artery is intercepted in the marginal zone (MZ) by macrophages and dendritic cells (DCs), as well as by a unique class of MZ B cells with innate-like behavior and limited BCR diversity (20). All three types of APCs are capable of conveying antigen to B cell follicles in the periarteriolar lymphoid sheaths (21), but certain types of antigens—especially repetitive bacterial polysaccharides and other T-independent type 2 antigens—are capable of activating MZ B cells directly (20) in a T-independent process (22, 23). In lymph nodes, antigen enters the subcapsular sinus (SCS) *via* afferent lymphatics and can reach B cell follicles in soluble form in the case of small antigens (<70 kDa) by movement through a conduit system that permeates the follicles (24, 25), or, for larger antigens and immune complexes, which are typically opsonized by complement components, intercepted by complement receptors on a layer of SCS macrophages (SSMs) lining the follicular (FO) zone, and then passed between complement receptors on various APCs and non-specific B cells. Immune complexes ultimately become tethered to the membrane of a follicular dendritic cell (FDC) (26, 27) for BCR scanning.

The BCR is composed of a membrane-bound immunoglobulin (mIg) for antigen binding and a transmembrane Igα/Igβ heterodimer for signaling (28). The mIg consists of two immunoglobulin light (L) chains and two heavy (H) chains, which have variable numbers hydrophobic amino acid sequence motifs in their cytoplasmic tails, depending on the Ig isotype. Antigen recognition is mediated by the hypervariable regions of mIg V_H_ and V_L_ segments, which fold to form an antigen-binding site; signaling is mediated by the cytoplasmic immunoreceptor tyrosine activation motifs (ITAMs) of the associated Igα/Igβ heterodimer. The spatial organization of BCRs on resting B cell surfaces and the effect of antigen engagement on this organization are incompletely understood. An early study showed by transmission electron microscopy that almost all plasma membrane-associated proteins, including BCRs, are present in clusters termed “protein islands” (29). Recently, point localization-based, super resolution fluorescence microscopy has provided information on the nanoscale spatial organization of BCRs on B cell surfaces at the level of individual BCRs. The results of three such studies (30–32) are consistent with models in which BCRs exist as monomers and in protein islands, and antigen encounter induces the coalescence of these into active signalosomes (33). By contrast, the results of Maity et al. (34) were interpreted to be consistent with a model in which BCRs exist in clusters on resting B cell surfaces that are disrupted by antigen resulting in the initiation of signaling (35). Clearly much remains to be learned about the nanoscale organization of BCRs that will add to our understanding of the initiation of BCR signaling.

Ultimately, microclusters of BCR with bound antigen and other co-receptors visible by diffraction-limited light microscopy form and encounter the intracellular tyrosine kinase Lyn. Lyn phosphorylates ITAMs on Igα and Igβ chains in BCR microclusters, providing a docking site for the tyrosine kinase Syk which initiates intracellular signaling cascades that allow the B cell to internalize antigen (36) [see Internalization of BCR and Intersection with MHCII in the MHCII Compartments (MIICs)]. Evidence from high-resolution total internal reflection microscopy in conjunction with fluorescence resonance energy transfer in living B cells argued that newly formed BCR microclusters perturbed the local lipid environment leading to the association of microclusters with a lipid raft probe and that this association facilitated the recruitment of Lyn to the BCR microclusters (37).

Soluble antigens are capable of initiating BCR clustering, but membrane-tethered antigens are more effective at inducing responses *in vivo* (38). This points to a critical role for FDCs and their use of long-term non-degradative compartments to store and recycle immune complexes and serve as an antigen depot (27). SSMs may also play a role in antigen presentation by conveying opsonized antigen directly to B cells after intercepting it in the SCS (38). Cell biological data indicate that APC/B cell interaction involves two major features. First, once stimulated, the B cell exhibits a membrane spreading and contraction response that assists with antigen aggregation and BCR microcluster formation and results in the formation of an immunological synapse with the APC (39, 40). Second, as the B cell separates from the APC, it extracts the target antigen from the surface of its partner cell.

The membrane-spreading response is accomplished within minutes of antigen contact by structural changes in the actin cytoskeleton. These changes involve cofilin-mediated severing of F-actin throughout the cell, followed by actin repolarization in the direction of the APC (41). The severing is thought to increase the mobility of cell-surface BCRs, assisting with formation of BCR/antigen microclusters and their movement into the center of the newly formed synapse (41, 42). The subsequent spreading response involves Arp2/3, resembling cell membrane extension of lamellipodia, whereas contraction requires dyneins and ERM proteins (40, 43). The microtubule cytoskeleton also changes shape in response to stimulation, with the microtubule-organizing center migrating rapidly toward the site of contact, in a process dependent on CDC42 and PKCζ (44).

When contraction is complete, the synapse adopts a form with a central supramolecular activation complex (cSMAC), containing a large cluster of antigen-bound BCRs surrounded by a ring of actin and adhesion molecules, the peripheral SMAC (pSMAC) that tethers the B cell to the APC (43, 45). B cell extraction of antigen from the cSMAC is accomplished by mechanical force, in which B cells physically pull on synaptic antigen through the BCR and deform flexible membrane substrates to promote antigen internalization (46, 47). B cells also can extract antigen by release of degradative MHCII-containing vesicles, containing lysosomal proteases and lipases, into the pSMAC (44). Recent evidence suggests that the mechanism of antigen extraction is dependent on the properties of the APC (48). The presence of MHC class II in the synapse along with lysosomal enzymes raises the possibility of peptide loading inside the synapse.

Due to the high affinity of their antigen receptor, B cells expressing specific BCRs require 1,000–10,000× less antigen to stimulate T cells compared to non-specific B cells (49). BCR affinity affects the efficiency of antigen collection from the surface of presenting cells (39), with contractile forces in the presenting cell setting a BCR affinity threshold for successful antigen capture (46). After ligand binding, high-affinity BCR forms larger microclusters for recruitment of signaling machinery (50). Lower affinity BCR also bind and internalize antigens, if a BCR avidity threshold, presumably required for clustering and its consequences, is surpassed (51), as can occur with multivalent antigens, particularly at the surface of presenting cells (52).

Cross-linking of BCR by multivalent ligands initiates B cell activation for antigen presentation. Evidence for activation by monovalent antigen has also emerged (31), with data suggesting a threshold for signaling that must be overcome by monovalent antigen to lead to antigen presentation. Membrane-bound monovalent antigen is apparently more effective than soluble for reaching this threshold [reviewed in Ref. (53)]. For naïve B cells, monovalent antigens can activate IgM-BCR, but immune complexes are required for stimulation of IgD-BCR (35).

## Internalization of BCR and Intersection with MHCII in the MHCII Compartments (MIICs)

After antigen binding, B cells internalize antigens mainly through clathrin-dependent, BCR-mediated endocytosis (46, 54). Clathrin-mediated endocytosis involves formation of clathrin-coated pits (CCPs), which pinch off plasma membrane to form intracellular vesicles. CCP formation is initiated by the phosphorylation of the adaptor, AP-2, which co-localizes with BCR/antigen clusters (55). The BCR binds AP-2 through its Igαβ subunits, the membrane-proximal tyrosine of a YxxØ (Ø = bulky hydrophobic) motif on the Igβ being the most important residue for receptor internalization (56). Intracellular vesicle generation requires actin polymerization and myosin activity (57, 58). After vesicle formation, clathrin disassociates, and the vesicle undergoes a cascade of biochemical changes, evolving into an early endosome and then into a late endosome. While most early endosomes are recycled back to the plasma membrane by Rab4, BCR signaling increases the rate of sorting into late endosomes *via* ubiquitination of BCR by c-Cbl and Cbl-b (59). Rab7 shepherds the transition of early endosome to late and appears to be directly modulated by BCR signaling (60). The final stage is delivery of vesicles carrying the antigen-bound BCR to peptide-loading compartments.

Reagents that cross-link the BCR and initiate signaling are 10× more efficient in delivering BCR to MHCII peptide-loading compartments than monovalent reagents (61). Lyn kinase activity associated with BCR signaling directly enhances endocytosis *via* phosphorylation of the clathrin heavy chain, which promotes its association with actin-coupling clathrin light chains (62, 63); BCR signaling also results in tyrosine phosphorylation of CCP-associated proteins (epsin15, epsin2, etc.) and downstream proteins (e.g., Vav, Btk) that play roles in both signaling and endocytosis (60, 64). BCR signaling promotes receptor endocytosis, but the exact roles for Igαβ motifs remain elusive, as signaling and endocytosis compete for the same motifs (56). Indeed, some evidence supports a model in which distinct BCR/antigen complexes mediate signaling and antigen internalization (65). Internalization of BCR inhibits signaling, as localization in endosomal compartments reduces surface BCR and abrogates the activity of signaling proteins, such as Lyn, Syk, and BLNK (66).

Endocytosed BCR/antigen complexes intersect with newly synthesized MHCII en route to the cell surface. Nascent MHCII αβ heterodimers are generated in the endoplasmic reticulum (ER) and rapidly associate with trimers of the type II membrane glycoprotein, Ii (CD74), a dedicated MHCII chaperone. Interaction with Ii facilitates MHCII folding and inhibits ligand binding to class II in the ER. Ii contains a peptide sequence (aa 81–104), referred to as the class II-associated invariant-chain peptide (CLIP) region, which non-covalently binds the MHCII groove. Four Ii isoforms exist: p41 and p43, which are expressed at low levels in B cells compared to other APC, and p33 and p35. p35 associates with clathrin-associated adaptor proteins; however, if all Ii species are p33, the complex can exit the ER as a pentamer (67). Export out of the ER occurs when all the retention motifs present in the trimer are “cancelled” (68). Trafficking of egressed MHCII–Ii complex, first to the trans-Golgi network, is controlled by cytosolic sorting tags. Recognition by AP-2 of two dileucine motifs in the Ii cytosolic domain enables αβIi sorting to clathrin-coated vesicles, and eventual localization to MIICs (69, 70). The exact definition of MIIC has been a matter of debate, but these compartments can be broadly characterized as late endosomal structures enriched in proteolytic enzymes and disulfide reductases and with sufficiently low pH to activate proteases involved in Ii degradation (see below) and antigen processing (54). The compartments exist in a several forms—multivesicular bodies (MVBs) or multilamellar structures—that are generally LAMP-1 positive (71).

Many Ii-mediated events are common to professional MHCII-expressing APC. However, lack of Ii in B cells and DCs in Ii-deficient mice shows some differential impact on MHCII-restricted antigen presentation. Some Ii-deficient DCs undergo normal maturation and present a broad range of peptidic epitopes from antigen internalized by fluid-phase endocytosis (72). By contrast, Ii is required for presentation of BCR-internalized antigen (73), and Ii-deficient B cells failed to present Ag-generated epitopes unless these epitope peptides were exogenously supplied (74), consistent with findings that, in B cells, Ii controls the transport of MHCII to and from MIIC (75) where BCR-delivered antigens are concentrated.

The intersection of antigen-bound BCR and MHCII appears to be actively promoted, as BCR binding by Fab′2 is associated with movement of MHCII to LAMP-1+ MVB vesicles (76) and ligated BCR and Ig-α/Ig-β also localize to these compartments, in a ubiquitin-dependent manner (77). Interaction of internalized BCR/antigen with intracellular MHCII was observed in pulldowns using biotin-labeled antigen (78). Strikingly, a rare (10%) conformation of MHCII seems to be a predominant conformer in complex with the BCR/antigen. This conformer (defined by the Ia.2 epitope) is highly effective for T cell activation, is enriched in lipid rafts, and stimulates intracellular calcium signaling in B cells (79, 80) Selectivity of the BCR/antigen for this MHCII conformer may contribute to the efficiency of BCR-mediated antigen presentation.

## Generation of the MHCII/Peptide Repertoire: Proteases and Regulation of Peptide Loading

Proteolytic events relevant for MHCII antigen presentation in B cells include processing of Ii and processing of endogenous and exogenous antigens, including BCR/antigen (81). Key enzymes implicated in epitope generation for MHCII presentation include lysosomal proteases that can be classified based on their catalytic mechanism into four major families (Table 1): (1) the cysteine cathepsins with a papain-fold (B, C, F, H, K, L, O, S, V, W, and X); (2) the aspartyl cathepsins D and E; (3) the serine cathepsins A and G, and (4) single member family of asparagine endopeptidase (AEP), a cysteine protease grouped with the caspases (82). In addition, gamma-interferon-inducible lysosomal thiol reductase (GILT) cleaves protein disulfide bonds (83). Cathepsins are synthesized as inactive zymogens that get modified by addition of mannose-6-phosphate residues (M6P), allowing recognition by M6P receptors in the trans-Golgi network and delivery to endosomal/lysosomal compartments. Cathepsin activation requires catalysis by other lysosomal proteases or by autocatalysis under certain circumstances, such as low pH or presence of glycosaminoglycans. Lysosomal proteases of the cysteine type have optimal activity at acidic pH, but other proteases are active over a range of pH. Inactivation occurs by protease degradation or by oxidation. Levels of expression and activity of cathepsins are controlled by multiple factors, including mRNA levels, local pH, and other protein factors that influence cathepsin trafficking and activation. For example, endogenous inhibitors like cystatins, stefins, tyropins, and serpins also regulate cathepsin function, although it appears that cystatins do not control antigen presentation (84).

**Table 1:** **Lysosomal proteases involved in MHC class II antigen processing**.

Lysosomal protease	Type/activity	Distribution in immune cells	Involved in (reference)
Cathepsin B	Cysteine/endo- and exopeptidase (carboxydipeptidase)	BCLs, DCs, and macrophages	Processing of immune complexes internalized *via* FcγR (85)
Cathepsin F	Cysteine/endopeptidase	Macrophages	Ii processing in absence of Cat S and L (86)
Cathepsin H	Cysteine/endo- and exopeptidase (aminopeptidase)	Ubiquitously expressed	Octapeptid (mini-chain) confers aminopeptidase activity; without the mini-chain function similar to Cat S (87)
Cathepsin S	Cysteine/endopeptidase	BCLs, DCs, macrophages, and epithelial cells	Converting the 10-kDa SLIP fragment from Ii into CLIP; processing of exogenous and endogenous antigen (88)
Cathepsin V or L2 (human homolog of mouse cathepsin L)	Cysteine/endopeptidase	Cortical human thymic epithelial cells; testis	Ii proteolysis in thymus (89)
Cathepsin D	Aspartic/endopeptidase	Ubiquitously expressed; also secreted	Destructive processing of myoglobin (90); there is evidence of redundancy among the aspartic cathepsins (Cat D and E)
Cathepsin E	Aspartic/endopeptidase	BCLs, B cells upon activation, monocyte-derived DCs, myeloid DCs, Langerhans cells	Processing ovalbumin; processing of TTC (91); processing of myoglobin (90); there is evidence of redundancy among the aspartic cathepsins (Cat D and E)
Cathepsin A	Serine/exopeptidase (carboxypeptidase)	Widely expressed, including primary B cells, BCLs, myeloid DCs, plasmacytoid DCs (92)	Removes amino acids from proteins or peptides C-terminal; may be important for fine trimming of peptides (92)
Cathepsin G	Serine/endopeptidase	Neutrophils, mast cells, B cells (endogenous/exogenous source), monocytes, myeloid DCs, plasmacytoid DCs; granule associated	Destructive processing/processing of MBP in primary B cells (93)
Asparagine endopeptidase	Cysteine/endopeptidase	BCLs; low in B cells; thymic CD11c+ DCs; F4/80+ thymic macrophages	Initial processing of Ii chain (94); *in vitro* intracellular processing of TTC (95); *in vitro* destructive processing of MBP (96, 97); not necessary for TTC processing *in vivo* (98); processing of Cat B, H, and L (99)

*BCLs, B cell lymphoblastoid line; DCs, dendritic cells; Ii, invariant chain; SLIP, small-leupeptin-induced protein; CLIP, class II-associated invariant-chain peptide; TTC, tetanus toxin, C-fragment; MBP, myelin basic protein*.

Proteolytic processing of Ii is required for loading of antigen peptides onto MHCII. Ii processing, which starts with formation of a 22 kDa leupeptin-induced protein, can be initiated by AEP (94). The second intermediate, 10 kDa fragment small-leupeptin-induced protein (SLIP) is then cleaved to generate the 25aa CLIP fragment. Cat S is the key component for converting the 10 kDa fragment into CLIP. In the human thymus, Cat V or L2 (Cat L in mice) is the main protease converting SLIP to CLIP. Studies of B cells and DCs from animals deficient in Cat S showed accumulation of surface MHCII with the 10 kDa Ii fragment (100), and reduced antigen presentation. In mice, in the absence of Cat S and L, Cat F in macrophages can generate CLIP, indicating cathepsin redundancy (82).

The crystallizable fragment (Fc) and antigen-binding fragment (Fab) domains of immunoglobulin remain largely intact in the MIIC of B cells (101, 102). Notably, however, the two domains can be immunoprecipitated separately, and co-precipitation of antigen with the Fab domain can be detected (101). This finding implies that proteolytic enzymes resident in the MIIC cleave intact immunoglobulin. In bone marrow-derived APC (over 80% macrophages), Cat B appears important for degradation of immunoglobulins that are taken up by Fc receptors (85).

Characterization of lysosomal proteases in B cells is still incomplete. Furthermore, one shortcoming of some studies on B cell proteases is the use of B lymphoblastoid cell lines (B-LCL), which may not reflect the endo/lysosomal environment in primary B cells. For example, B-LCL degrade myelin basic protein (MBP) by AEP, but human primary B cells have low level of AEP activity and instead use exogenous Cat G (103), a granule-associated protease that binds the B cell surface *via* a thrombin-like receptor and is internalized into endosomes (93). In addition, human B-LCL contain active CatS, CatB, and CatD, whereas none of these cathepsins are detected in resting primary human B cells (103). Cat E expression is enhanced in primary tonsillar B cells after activation with *Staphylococcus aureus* (104); similarly, PMA activation increases expression of Cat E in primary B cells from blood and increases presentation of tetanus toxin C-fragment to T cells (91). Thus, some of the differences between B-LCL and primary B cell lysosomal protease content may be due to activation state. Murine primary B cells express lysosomal GILT, and GILT activity decreases Cat S expression and function (105), suggesting that GILT participates in control of cathepsin activity in B cells.

The cathepsin profile of B cells has similarities to the DC profile (Table 1). B cells and DCs have lower levels of lysosomal proteases, with corresponding lower proteolytic activity, compared to macrophages. These differences appear to be specific to the lysosomal proteases and cannot be ascribed to number of lysosomes. Although the rate of antigen internalization is similar between DCs and macrophages, the rate of proteolysis is slower in DCs. For test antigens, protection from degradation is associated with enhanced presentation to T cells (106).

Studies of mice deficient for specific proteases show that proteases can influence the repertoire of peptides presented by MHCII. However, it is largely unknown if these effects are mediated by direct participation of specific proteases in endosomal antigen processing or predominantly influence Ii processing. For example, a series of studies showed that in B cells, as well as in DCs, Cat S does not directly influence the generation of most peptides presented by MHCII, but some peptides are affected [reviewed in Ref. (88)]. An additional challenge is that degradation of a given protein may require a series of proteases. For example, degradation of MBP *in vitro* is initiated by AEP and Cat S, and intermediates are subsequently degraded by other proteases including Cat S, D, and L (96). Data from an *in vitro* antigen-processing model system using only three cathepsins (Cat B, H, and S) argued that sensitivity to these cathepsins, the structure of proteins, strength of peptide binding to HLA-DR, and HLA-DM susceptibility (see below) were all factors that influence which peptides are presented by MHCII (107). This *in vitro* system recapitulated generation of naturally processed peptides from test proteins, suggesting its potential value for epitope prediction and for modeling mechanistic steps in antigen processing for MHCII cargo (108). Determination of the *in vivo* roles for specific proteases remains a subject of on-going work.

Availability of antigen for processing is influenced BCR affinity. Higher affinity binding allows delivery of protected regions to later compartments for peptide generation and binding to nascent MHCII (although highest affinity binding may suppress epitope availability), while lower affinity allows release of antigen in earlier compartments and generation of peptides that can bind recycling MHCII (109). For certain antibodies with highly pH-dependent binding to antigen, lower pH of the later compartments may greatly facilitate epitope generation (110). BCR binding suppresses some and enhances availability of other T cell epitopes, with both types of determinants within the region of the antigen “footprinted” by the antibody (111). BCR-bound antigen can persist in B cells, providing a source of peptide longer than antigen taken up by fluid phase (112).

Like other professional APCs, B cells express a non-classical class II molecule, HLA-DM (DM; H2-M in mice), with highest concentrations in late endocytic compartments. Despite the structural similarity with classical MHCII, DM does not directly bind peptides (113); instead, it modulates the peptidome *via* a catalytic mechanism (114, 115). DM function is crucial; DM-null APCs are defective in MHCII antigen processing and presentation (116) and DM-deficient mice present a skewed peptide repertoire, impairing selection of CD4+ T cells (117). The generally accepted function of DM is that it stabilizes a peptide-receptive form of MHCII by a transient association (118, 119); and this temporary DM/MHCII interaction facilitates the removal of weak binding peptides, such as CLIP and cryptic epitopes, while promoting the loading of high-affinity peptides that mostly become immunodominant epitopes for MHCII presentation at the surface of B cells (120–123). DM-regulated epitope selection (DM editing) is a pH-dependent process with pH optima (4.5–5.5), recapitulating late endosomal–lysosomal conditions in MIICs (124, 125).

Another non-classical class II molecule, HLA-DO (DO), also plays an essential role in antigen presentation in B cells (126–129), as knockout of the murine DO homolog, H2-O, leads to immunodeficiency and autoimmunity that are directly related to dysfunction of antigen presentation by bone marrow-derived cells (130). Although DO is structurally quite homologous to classical MHCII, there is no evidence that it can directly present peptides. However, DO mimics MHCII in binding to the same site on DM, such that it blocks the catalytic action of DM on MHCII/peptide complexes (113, 131). The requirement for DM inhibition by DO in antigen presentation in a restricted group of APCs (132) implies unique features of the MHCII peptidomes in these cells, especially B cells.

The affinity of DO/DM binding (nanomolar) (125, 133) is 1,000- to 10,000-fold stronger than that of the transient binding of MHCII to DM (micromolar) (131), and DO requires the tight association with DM to exit the ER for translocation to its lysosomal destination (134). The molecular interaction between intact DO and DM is pH insensitive, even at the lysosomal pH (125). This raises questions as to how DO-associated DM is freed to edit MHCII-associated peptide cargo, and what role DO plays to modulate the B cell MHCII peptidome. The stoichiometry of DM:MHCII is a critical factor in the pH-dependence of DM editing (125). DM abundance can compensate for the reduction in its enzymatic activity at pH > 5.5, and this potentially allows DM editing in high DM-expressing APCs, like B cells, to emerge outside late endocytic MIIC. Therefore, DO likely serves as a regulator to control the abundance of free DM and keep DM function focused on acidic MIICs (also see GC B Cells).

Multiple mechanisms have been postulated to explain how DO/DM complexes facilitate peptide loading onto MHCII [details are reviewed in Ref. (129, 135–137)]. Recent evidence argues that DO/DM complexes are inert for peptide exchange catalysis, but that in lysosomal conditions, low pH promotes denaturation of DO within the complex to elevate the level of free, catalytically active DM (125, 133). pH-dependent DO removal is an irreversible process that may be associated with many antigen-triggered events in the B cell, including the acidification of lysosomes, BCR signal-mediated transcriptional modifications of MHCII or the class II transactivator (CIITA), and redistribution of MHCII proteins within a multivesicular MIIC. An alternative view, that DO optimizes presentation of DM-resistant peptides through direct interaction with a peptide-receptive form of MHCII, has been presented (138).

The cytoplasmic tails of both DM and DO contain lysosomal targeting motifs. Interestingly, without DO, DM can reside outside of lysosomes, for instance, on the surface of immature myeloid DCs (139) or on secreted exosomes (140). Although no high-resolution imaging data are available, these studies at least suggest that the sorting motif on DO constrains the intracellular location of DO/DM complexes to lysosomes, where DM is further distributed away from internal membranes (source of exosomes) to the limiting membrane (140, 141). As (usually foreign) antigens associated with BCRs are typically protected from proteolysis at early endosomal pH, DM editing likely is unnecessary at the earliest stages of antigen endocytosis in B cells. Lysosome-oriented trafficking together with functional inhibition conferred by DO would focus DM editing on epitopes derived from BCR/antigen complexes that also localize to MIICs. DO-regulated trafficking may rely on the ubiquitination of cytoplasmic tails of DO heterodimers by, for example, a MARCH family E3 ligase (142).

The ectodomain of DM has reduced activity at pH 4.6 likely due to denaturing of free DM (125). This implies that DO may reciprocally chaperone and stabilize DM through tight association and maintain DM levels in lysosomes; this effect is also suggested by evidence in DO-expressing DCs from transgenic mice (143). Such bi-directional chaperoning would persist until an antigen exposure drives DO removal in MIIC. In agreement with this hypothesis, several studies have shown a concomitant decline of DM and DO during B cell activation (126, 127), although the DM:DO ratio increases in favor of DM editing.

**Figure 1:**
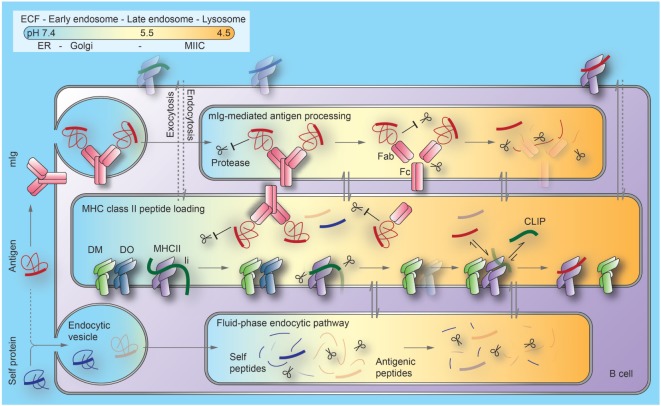
**Current model of the regulation of antigen processing and presentation in B cells**. B cell antigen uptake by membrane immunoglobulin (mIg) is substantially (1,000×) more efficient than by fluid-phase endocytosis. Binding to mIg protects regions of antigens from proteolysis at the early stages of the endocytic pathway. As pH decreases along this pathway, Ig can be processed into antigen-binding fragment (Fab) and crystallizable fragment (Fc) domains. Antigens become available for peptide generation through a combination of unfolding, proteolytic processing, and release from Ig. MHCII/invariant chain (Ii) complexes, DM and its inhibitor, DO, are generated in the endoplasmic reticulum (ER) and transported through the Golgi to late endosomal compartments, termed MIIC. Although Ii processing begins as MHCII/Ii complexes traverse earlier endocytic compartments, exchange of the final Ii remnant peptides (CLIP) bound to the MHCII peptide binding groove with antigenic peptides or self-peptides is inefficient before MHCII enters MIIC. Mechanisms limiting peptide exchange until this stage include (1) DO significantly inhibits DM catalysis of peptide loading and (2) Fab-associated antigen fragments are largely protected from proteolysis to peptides. At late stages of these pathways, lysosomes merge with MIIC. This generates the highly acidic condition that favors denaturation and degradation of DO and Fab, and DM-mediated peptide exchange. Most derivatives of self-proteins internalized without mIg cannot survive endosomal/lysosomal proteolysis. Arrows indicate protein trafficking through different intracellular compartments (indicated by colors) along each pathway. Dotted lines indicate unfavorable processes. ECF, extracellular fluid; MHCII, major histocompatibility complex class II proteins; MIIC, MHCII compartments; CLIP, class II invariant-chain-derived peptides.

As DO/DM and BCR/antigen complexes meet in MIICs, DM may also function in facilitated antigen hand-off from BCR to MHCII proteins. The recent findings that DM and mIg co-precipitate from cell lysates and that soluble recombinant DM and Fab fragments (but not Fc fragments) co-precipitate *in vitro* raise the possibility of a multimeric class II peptide-loading complex, including membrane Ig, DM, and MHC proteins (144). Figure 1 summarizes key events described in Section “Generation of the MHCII/Peptide Repertoire: Proteases and Regulation of Peptide Loading” of the review.

## MHCII/Peptide Complex Transport to the Surface

The molecular details of transport of newly loaded MHCII/peptide complexes from the MIIC to the cell surface are not delineated in mechanistic detail for any APC, although clearly CLIP replacement with other peptides is not a requirement for egress from MIICs. More is known about the regulation of MHCII surface expression and its relationship to recycling of newly synthesized MHCII in complex with peptide. Available data argue that B cell activation does not influence the endocytosis of surface MHCII, yet it clearly alters the level of MHCII expression. Cho et al. demonstrated that internalized MHCII efficiently recycles in activated B cells, but not in resting B cells. The lack of MHCII recycling in these cells is a consequence of oligoubiquitination of a single conserved lysine (K225) in the C-terminal cytosolic tail of the MHCII β chain by the E3 ubiquitin ligase MARCH1. Further, MHCII ubiquitination promotes sorting to MVB and turnover of MHCII in lysosomes. Upon antigen ligation of BCRs, MARCH1 expression and MHCII ubiquitination is halted, enabling MHCII escape from degradation, rapid recycling, and increased surface expression. The activation induced boost in MHCII also promotes the ability of B cells to present the activating antigens (145).

## MHCII Antigen Presentation Over the Span of B Cell Development

### Early B Cell Development

**Figure 2:**
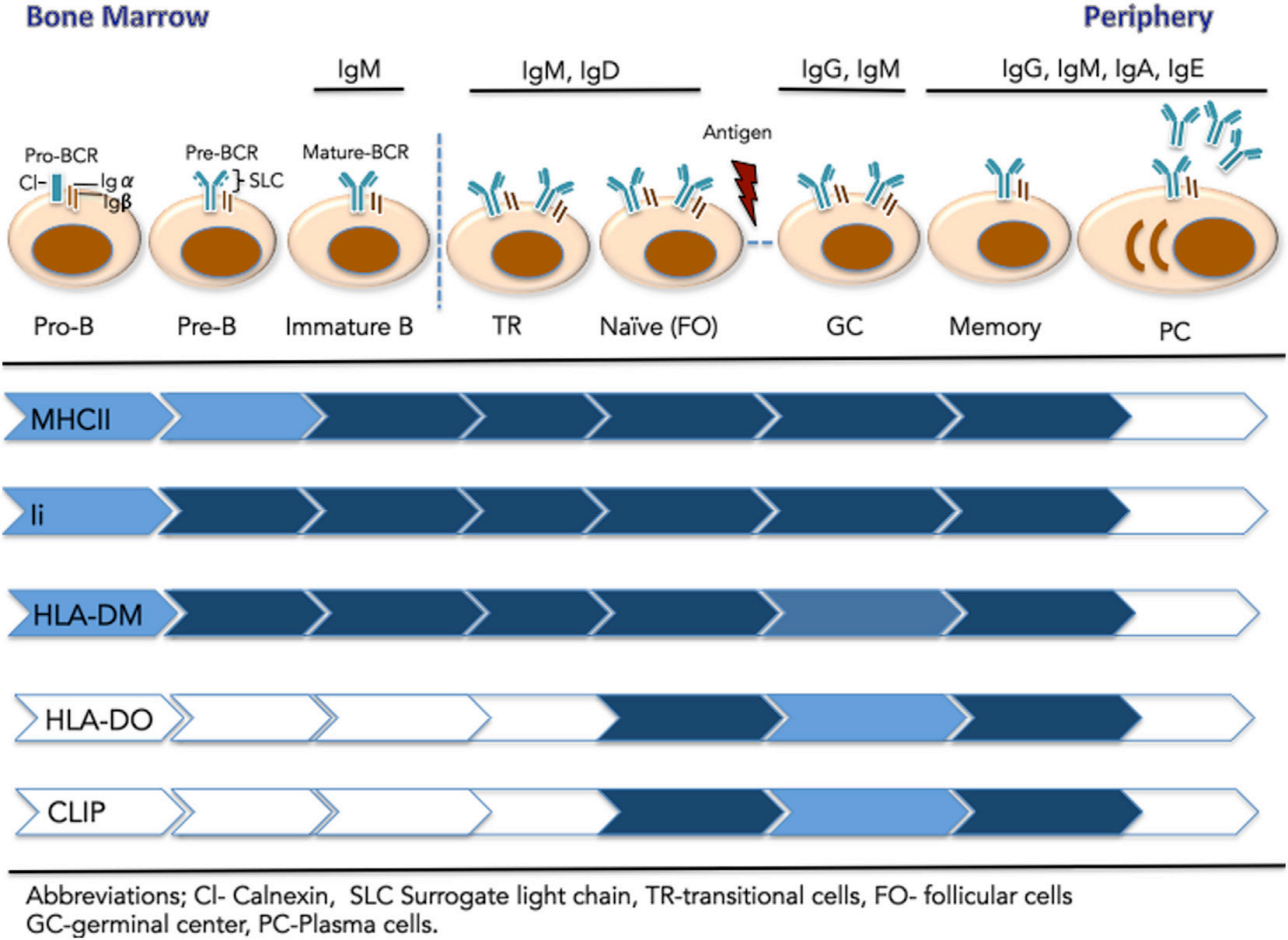
**Expression of components of the major histocompatibility complex class II (MHCII) antigen presentation pathway across the stages of B cell development**. Early B cells (pro-B, pre-B, and immature-B-cell) reside in the bone marrow. These stages are defined by the rearrangement of V, D, and J gene segments at the Ig heavy and light chain loci. At the pre-B cell stages, the pre-BCR is composed of a heavy chain paired with the surrogate light chain (λ 5-Vpre-B), together with signaling components Ig-α and Ig-β. The mature IgM-B cell receptor (BCR) emerges on immature B cells that exit the BM. They migrate to the spleen as transitional B cells and mature to naïve, pre-immune cells of the follicular (FO) B subset, found in the blood and secondary lymphoid organs. Upon immunological challenge, naïve FO B cells that successfully process and present MHCII-bound antigen to CD4+ T cells, differentiate into centroblasts and centrocytes organized in germinal centers. Following BCR affinity maturation, selected B cells further differentiate into long-lived memory B cells or antibody-secreting plasma cells. Relative expression of the MHCII antigen presentation machinery components is represented by color intensity.

Early stages of B cell development in the bone marrow (pro- and pre-B cells) are defined by stepwise rearrangement of V, D, and J gene segments at the Ig heavy and light chain loci [(146); see Figure 2]. The first BCR complex, expressed at the pre-B cell stage, is composed of the heavy-chain gene product with a surrogate light chain (λ 5-Vpre-B), together with the Igα/Igβ heterodimer (147, 148). Interestingly, antigen-presenting machinery is already present at the pro- and pre-B stages, as CIITA is expressed and drives expression of MHCII, HLA-DM, and Ii (149, 150). The presence of most MHCII pathway components (except DOβ) in the absence of mature BCR raises the question of their potential function in B cell development. Strikingly, the Ii N-terminal domain (aa 1–82) has an alternate activity in B cells that does not relate to its role in antigen presentation. Ii-deficient mice show an arrest in B cell development with an enlarged population of immature IgDlow CD23low transitional B cells. When the Ii N-terminal domain alone is expressed in transgenic mice, MHCIi trimer formation and egress from the ER is recovered, but MHCII/peptide formation and surface expression is not restored (151). Rather, this Ii domain initiates a signaling cascade, resulting in NFkB activation and B cell maintenance (152). The intramembrane protease SPPL2a (presenilin homolog signal-peptide-peptidase-like 2a) catalyzes the generation of this Ii fragment in mice and human B cells (153) Ii has third role for B cells as the receptor for macrophage migration inhibitory factor and interaction leads to signaling cascade that promotes B cell chemotaxis and survival (154).

In contrast to effects of Ii knock out, B cell development is only subtly altered in MHCII-deficient mice. In these mice, a mature B cell population is established, although the overall number of mature B cells and their capacity for antigen presentation is impaired (155, 156). B cells also reach the mature stage in bare lymphocyte syndrome patients, who lack MHCII expression due to mutation of CIITA (157). In DM-deficient mice, B cells mature, but display a reduced response to antigen (117). In light of these results, Chen and Jensen (156) proposed that the presence of the class II antigen presentation machinery in pre-, pro-, and immature BM B cells serves as a mechanism to induce peripheral CD4+ T cell tolerance through presentation of peptides from self-antigens (156). Low or no MHCII/CLIP complexes are detected on these B cells, implying that MHCII undergoes at least one peptide exchange cycle to remove CLIP and bind self-peptides. This possibility is consistent with expression of DM without DO at these early stages. As CD40 is not expressed on immature B cells, recognition by autoreactive T cells would likely result in anergy or deletion. Additional work is needed to confirm this hypothesis.

### Immature Transitional and Naïve B Cells

Following BCR maturation, IgM is expressed on the surface of immature B cells that exit the BM and migrate to the spleen as transitional B cells (12). Close to 85% of the original B cell repertoire is eliminated by negative selection of cells expressing autoreactive BCR. Transitional B cells further mature to naïve/pre-immune B cells of the FO B subset in blood and SLO or to MZ B cells of the splenic white pulp (158, 159). Expression of DO emerges in naïve (IgM+/IgD+/CD40+) B cells (142). DM levels are relatively stable throughout B cell development, leading to a high DO:DM ratio at this stage. This ratio inhibits DM activity, particularly in early, higher pH endosomal compartments, which are accessed by fluid-phase endocytosis of self-proteins. Reduced DM peptide exchange activity results in high levels of MHCII/CLIP complexes on naïve B cells. DO activity may thus limit the MHCII/self-peptide repertoire in naïve circulating B cells, reducing the possibility of recognition by autoreactive T cells (128, 160).

### GC B Cells

Presentation of MHCII/peptide by FO B cells in the lymph node mediates cognate interaction with CD4+ T cells, co-stimulated by CD40–CD40L interaction. Initial T cell contact drives B cell differentiation into centroblasts and centrocytes organized in GCs, with B cell-rich dark zones, where centroblasts undergo somatic hypermutation, yielding clonal variants of GC B cells with modified antigen affinity and specificity (161). Centroblasts further differentiate into centrocytes and enter the GC light zone, where encounters with FDC reintroduces antigen to the modified BCR. Antigen uptake and presentation permits cognate interaction with T_FH_ cells, in proportion MHCII/peptide levels (4). Centrocytes with higher affinity for antigen are selectively preserved, whereas those with reduced BCR affinity are deleted. Additionally, a subset of centrocytes undergoes class-switch recombination for expression of IgG, IgA, or IgE (161–163). Reduced DO expression marks GC entry of B cells and is primarily driven by post transcriptional events, such as ubiquitination by MARCH9 (126, 127, 142). Centrocytes likely benefit from maximum DM editing both kinetically and stoichiometrically to achieve high levels of stable MHCII/peptide complexes and garner T cell help. Such a high level of DM activity cannot simply stem from transcriptional upregulation; indeed, GC B cells express slightly lower levels of DM protein than naïve or memory B cells (127). This suggests that the boost in DM editing originates from pre-existing inactive DM that was most likely associated with DO.

Major histocompatibility complex class II/peptide complexes on the surface of GC centroblasts are ubiquitinated and targeted for degradation, whereas centrocytes express long-lived MHCII/peptide complexes (164). The MHCII turnover in these cells is controlled by oligoubiquitination of the MHCII β chain cytosolic tail by MARCH1. MHCII ubiquitination prevents the accumulation of older MHCII/peptide complexes in GC centroblast B cells and allows antigen captured *via* affinity modulated BCR to be presented on newly expressed MHCII, thus reporting on the SHM process (165). Once cells move to the light zone as centrocytes, MHCII/peptide complexes on the cell surface are stabilized, facilitating productive interactions with CD4+ T cells during selection for high-affinity clones.

### Post GC Memory B and Plasma Cells (PC)

Centroblasts and centrocytes circulate between the light and dark zone until an optimal level of BCR affinity is achieved, and the selected B cells can further differentiate into long-lived memory B cells or antibody-secreting PC (161). Memory B cells express DO and DM and reestablish a higher DO:DM ratio compared to GC B cells. The model of irreversible DO removal (see Generation of the MHCII/Peptide Repertoire: Proteases and Regulation of Peptide Loading) need not conflict with the observation that memory B cells leaving the GC have upregulated DO levels (126, 127), given the rapid differentiation and cell division of phenotypically polarized GC B cells (166). Asymmetric segregation of proteins relevant for T cell interaction could allow polarized GC B cells to give rise to daughter cells with distinct fates: an effector-prone cell that bears most of free DM capable of antigen presentation to T cells, and a memory-prone cell that inherits most of the DO/DM complex and exits GC with high DO:DM ratio. Surface MHCII/CLIP levels on memory B cells are lower than levels on naïve cells (167); this finding could result from stability of MHCII/peptide complexes acquired during the GC stage.

Studies of mice lacking MHCII expression in memory cells showed impaired ability of these cells to differentiate into PC upon antigen restimulation (168). During the B memory response, B/T cell cognate interaction again promotes selective activation of affinity matured memory B cells that will yield PC secreting high-affinity antibodies. After B cell differentiation into PC, expression of CIITA, which controls most components of the class II processing pathway, is repressed, yielding marked reductions in the levels of DM, DOα, Ii, and MHCII (167). Though DOβ expression is not regulated by CIITA, the lack of DOα expression together with DO requirement for DM to egress from the ER results in loss of functional DO.

## Conclusion

Many essential molecular mechanisms modulating the protein dynamics of MHCII-restricted antigen presentation by B cells have come to light in recent years, revealing the elegance of this system for carefully regulated functions across the span of the B cell life cycle. In parallel (though not the focus of this review), emerging evidence indicates a critical role for B cell antigen presentation function in various immune and autoimmune reactions. Nonetheless, key questions remain to be answered. What initiates and regulates fluid-phase endocytosis in B cells and what is the role of this process in early B cells that lack mature BCR? What molecular events mediate independent and interdependent signaling and antigen internalization downstream of antigen binding to the BCR? How does antigen binding influence the architecture of the endocytic pathway and the composition and protease content of intracellular compartments? What factors in addition to pH influence the DM:DO ratios in endocytic compartments? What are the mechanisms involved transport to the surface of MHCII/peptide complexes? Are there unique features of the MHCII pathway in memory B cells? Undoubtedly, B cell-mediated MHCII antigen presentation is an exciting and important field to watch in the coming years.

## Author Contributions

LA, S-cH, MM, KB, CM, WJ, and EM contributed sections to this review. EM edited the full review and generated the final draft. LA, WJ, and CM contributed the tables and figures.

## Conflict of Interest Statement

The authors declare that the research was conducted in the absence of any commercial or financial relationships that could be construed as a potential conflict of interest.

## References

[1] Yamano T Nedjic J Hinterberger M Steinert M Koser S Pinto S Thymic B cells are licensed to present self antigens for central T cell tolerance induction. Immunity (2015) 42:1048–61. 10.1016/j.immuni.2015.05.013 26070482

[2] Constant SL. B lymphocytes as antigen-presenting cells for CD4+ T cell priming in vivo. J Immunol (1999) 162:5695–703. 10229801

[3] Bradley LM Harbertson J Biederman E Zhang Y Bradley SM Linton PJ. Availability of antigen-presenting cells can determine the extent of CD4 effector expansion and priming for secretion of Th2 cytokines in vivo. Eur J Immunol (2002) 32:2338–46. 10.1002/1521-4141(200208)32:8<2338::AID-IMMU2338>3.0.CO;2-R 12209647

[4] Gitlin AD Shulman Z Nussenzweig MC. Clonal selection in the germinal centre by regulated proliferation and hypermutation. Nature (2014) 509:637–40. 10.1038/nature13300 24805232 PMC4271732

[5] Shulman Z Gitlin AD Weinstein JS Lainez B Esplugues E Flavell RA Dynamic signaling by T follicular helper cells during germinal center B cell selection. Science (2014) 345:1058–62. 10.1126/science.1257861 25170154 PMC4519234

[6] Misumi I Whitmire JK. B cell depletion curtails CD4+ T cell memory and reduces protection against disseminating virus infection. J Immunol (2014) 192:1597–608. 10.4049/jimmunol.1302661 24453250 PMC3925510

[7] Bautista BL Devarajan P Mckinstry KK Strutt TM Vong AM Jones MC Short-lived antigen recognition but not viral infection at a defined checkpoint programs effector CD4 T cells to become protective memory. J Immunol (2016) 197:3936–49. 10.4049/jimmunol.1600838 27798159 PMC5113829

[8] Barnett LG Simkins HM Korn LL Johnson AL Wherry EJ B cell antigen presentation in the initiation of follicular helper T cell and germinal center differentiation. J Immunol (2014) 192:3607–17. 10.4049/jimmunol.1301284 24646739 PMC4380085

[9] Wong FS Wen L Tang M Ramanathan M Visintin I Daugherty J Investigation of the role of B-cells in type 1 diabetes in the NOD mouse. Diabetes (2004) 53:2581–7. 10.2337/diabetes.53.10.2581 15448087

[10] Molnarfi N Schulze-Topphoff U Weber MS Patarroyo JC Prod’homme T Varrin-Doyer M MHC class II-dependent B cell APC function is required for induction of CNS autoimmunity independent of myelin-specific antibodies. J Exp Med (2013) 210:2921–37. 10.1084/jem.20130699 24323356 PMC3865476

[11] Nakken B Munthe LA Konttinen YT Sandberg AK Szekanecz Z Alex P B-cells and their targeting in rheumatoid arthritis – current concepts and future perspectives. Autoimmun Rev (2011) 11:28–34. 10.1016/j.autrev.2011.06.010 21777703

[12] Naradikian MS Scholz JL Oropallo MA Cancro MP Understanding B cell biology. In: Bosch X Ramos-Casals M Khamashta MA, editors. Drugs Targeting B-Cells in Autoimmune Diseases. Basel: Springer Basel (2014). p. 11–35.

[13] Beland K Marceau G Labardy A Bourbonnais S Alvarez F. Depletion of B cells induces remission of autoimmune hepatitis in mice through reduced antigen presentation and help to T cells. Hepatology (2015) 62:1511–23. 10.1002/hep.27991 26175263

[14] Fraussen J Claes N Van Wijmeersch B van Horssen J Stinissen P Hupperts R B cells of multiple sclerosis patients induce autoreactive proinflammatory T cell responses. Clin Immunol (2016) 173:124–32. 10.1016/j.clim.2016.10.001 27717695

[15] Jackson SW Kolhatkar NS Rawlings DJ. B cells take the front seat: dysregulated B cell signals orchestrate loss of tolerance and autoantibody production. Curr Opin Immunol (2015) 33:70–7. 10.1016/j.coi.2015.01.018 25679954 PMC4397155

[16] Ghosn EE Waters J Phillips M Yamamoto R Long BR Yang Y Fetal hematopoietic stem cell transplantation fails to fully regenerate the B-lymphocyte compartment. Stem Cell Reports (2016) 6:137–49. 10.1016/j.stemcr.2015.11.011 26724903 PMC4720028

[17] Herzenberg LA. B-1 cells: the lineage question revisited. Immunol Rev (2000) 175:9–22. 10.1111/j.1600-065X.2000.imr017520.x 10933587

[18] Owen JA Punt J Stranford SA Jones PP Kuby J Kuby Immunology. New York: W.H. Freeman (2013).

[19] Rios D Wood MB Li J Chassaing B Gewirtz AT Williams IR. Antigen sampling by intestinal M cells is the principal pathway initiating mucosal IgA production to commensal enteric bacteria. Mucosal Immunol (2016) 9:907–16. 10.1038/mi.2015.121 26601902 PMC4917673

[20] Cerutti A Cols M Puga I Marginal zone B cells: virtues of innate-like antibody-producing lymphocytes. Nat Rev Immunol (2013) 13:118–32. 10.1038/nri3383 23348416 PMC3652659

[21] Kraal G. Cells in the marginal zone of the spleen. Int Rev Cytol (1992) 132:31–74. 10.1016/S0074-7696(08)62453-5 1555921

[22] Martin F Oliver AM Kearney JF. Marginal zone and B1 B cells unite in the early response against T-independent blood-borne particulate antigens. Immunity (2001) 14:617–29. 10.1016/S1074-7613(01)00129-7 11371363

[23] Puga I Cols M Barra CM He B Cassis L Gentile M B cell-helper neutrophils stimulate the diversification and production of immunoglobulin in the marginal zone of the spleen. Nat Immunol (2011) 13:170–80. 10.1038/ni.2194 22197976 PMC3262910

[24] Pape KA Catron DM Itano AA Jenkins MK. The humoral immune response is initiated in lymph nodes by B cells that acquire soluble antigen directly in the follicles. Immunity (2007) 26:491–502. 10.1016/j.immuni.2007.02.011 17379546

[25] Harwood NE Batista FD. The antigen expressway: follicular conduits carry antigen to B cells. Immunity (2009) 30:177–9. 10.1016/j.immuni.2009.01.004 19239901

[26] Batista FD Harwood NE. The who, how and where of antigen presentation to B cells. Nat Rev Immunol (2009) 9:15–27. 10.1038/nri2454 19079135

[27] Heesters BA Chatterjee P Kim YA Gonzalez SF Kuligowski MP Kirchhausen T Endocytosis and recycling of immune complexes by follicular dendritic cells enhances B cell antigen binding and activation. Immunity (2013) 38:1164–75. 10.1016/j.immuni.2013.02.023 23770227 PMC3773956

[28] Reth M. Antigen receptors on B lymphocytes. Annu Rev Immunol (1992) 10:97–121. 10.1146/annurev.iy.10.040192.000525 1591006

[29] Lillemeier BF Pfeiffer JR Surviladze Z Wilson BS Davis MM. Plasma membrane-associated proteins are clustered into islands attached to the cytoskeleton. Proc Natl Acad Sci U S A (2006) 103:18992–7. 10.1073/pnas.0609009103 17146050 PMC1681352

[30] Mattila PK Feest C Depoil D Treanor B Montaner B Otipoby KL The actin and tetraspanin networks organize receptor nanoclusters to regulate B cell receptor-mediated signaling. Immunity (2013) 38:461–74. 10.1016/j.immuni.2012.11.019 23499492

[31] Avalos AM Bilate AM Witte MD Tai AK He J Frushicheva MP Monovalent engagement of the BCR activates ovalbumin-specific transnuclear B cells. J Exp Med (2014) 211:365–79. 10.1084/jem.20131603 24493799 PMC3920557

[32] Lee J Sengupta P Brzostowski J Lippincott-Schwartz J Pierce SK The nanoscale spatial organization of B cell receptors on IgM- and IgG-expressing human B cells. Mol Biol Cell (2016) 28:511–23. 10.1091/mbc.E16-06-0452 27974642 PMC5305258

[33] Liu W Wang H Xu C. Antigen receptor nanoclusters: small units with big functions. Trends Immunol (2016) 37:680–9. 10.1016/j.it.2016.07.007 27555115

[34] Maity PC Blount A Jumaa H Ronneberger O Lillemeier BF Reth M. B cell antigen receptors of the IgM and IgD classes are clustered in different protein islands that are altered during B cell activation. Sci Signal (2015) 8:ra93. 10.1126/scisignal.2005887 26373673

[35] Hobeika E Maity PC Jumaa H. Control of B cell responsiveness by isotype and structural elements of the antigen receptor. Trends Immunol (2016) 37:310–20. 10.1016/j.it.2016.03.004 27052149

[36] Dal Porto JM Gauld SB Merrell KT Mills D Pugh-Bernard AE Cambier J. B cell antigen receptor signaling 101. Mol Immunol (2004) 41:599–613. 10.1016/j.molimm.2004.04.008 15219998

[37] Sohn HW Tolar P Pierce SK. Membrane heterogeneities in the formation of B cell receptor-Lyn kinase microclusters and the immune synapse. J Cell Biol (2008) 182:367–79. 10.1083/jcb.200802007 18644892 PMC2483512

[38] Carrasco YR Batista FD. B cell recognition of membrane-bound antigen: an exquisite way of sensing ligands. Curr Opin Immunol (2006) 18:286–91. 10.1016/j.coi.2006.03.013 16616474

[39] Fleire SJ Goldman JP Carrasco YR Weber M Bray D Batista FD. B cell ligand discrimination through a spreading and contraction response. Science (2006) 312:738–41. 10.1126/science.1123940 16675699

[40] Yuseff MI Pierobon P Reversat A Lennon-Dumenil AM. How B cells capture, process and present antigens: a crucial role for cell polarity. Nat Rev Immunol (2013) 13:475–86. 10.1038/nri3469 23797063

[41] Freeman SA Lei V Dang-Lawson M Mizuno K Roskelley CD Gold MR. Cofilin-mediated F-actin severing is regulated by the Rap GTPase and controls the cytoskeletal dynamics that drive lymphocyte spreading and BCR microcluster formation. J Immunol (2011) 187:5887–900. 10.4049/jimmunol.1102233 22068232

[42] Hao S August A. Actin depolymerization transduces the strength of B-cell receptor stimulation. Mol Biol Cell (2005) 16:2275–84. 10.1091/mbc.E04-10-0881 15728723 PMC1087234

[43] Kuokkanen E Sustar V Mattila PK. Molecular control of B cell activation and immunological synapse formation. Traffic (2015) 16:311–26. 10.1111/tra.12257 25639463

[44] Yuseff MI Reversat A Lankar D Diaz J Fanget I Pierobon P Polarized secretion of lysosomes at the B cell synapse couples antigen extraction to processing and presentation. Immunity (2011) 35:361–74. 10.1016/j.immuni.2011.07.008 21820334

[45] Carrasco YR Fleire SJ Cameron T Dustin ML Batista FD. LFA-1/ICAM-1 interaction lowers the threshold of B cell activation by facilitating B cell adhesion and synapse formation. Immunity (2004) 20:589–99. 10.1016/S1074-7613(04)00105-0 15142527

[46] Natkanski E Lee WY Mistry B Casal A Molloy JE Tolar P. B cells use mechanical energy to discriminate antigen affinities. Science (2013) 340:1587–90. 10.1126/science.1237572 23686338 PMC3713314

[47] Nowosad CR Spillane KM Tolar P. Germinal center B cells recognize antigen through a specialized immune synapse architecture. Nat Immunol (2016) 17:870–7. 10.1038/ni.3458 27183103 PMC4943528

[48] Spillane KM Tolar P. B cell antigen extraction is regulated by physical properties of antigen-presenting cells. J Cell Biol (2017) 216:217–30. 10.1083/jcb.201607064 27923880 PMC5223605

[49] Lanzavecchia A Bove S. Specific B lymphocytes efficiently pick up, process and present antigen to T cells. Behring Inst Mitt (1985) (77):82–7. 3002320

[50] Liu W Meckel T Tolar P Sohn HW Pierce SK. Antigen affinity discrimination is an intrinsic function of the B cell receptor. J Exp Med (2010) 207:1095–111. 10.1084/jem.20092123 20404102 PMC2867278

[51] Barral P Eckl-Dorna J Harwood NE De Santo C Salio M Illarionov P B cell receptor-mediated uptake of CD1d-restricted antigen augments antibody responses by recruiting invariant NKT cell help in vivo. Proc Natl Acad Sci U S A (2008) 105:8345–50. 10.1073/pnas.0802968105 18550831 PMC2448839

[52] Suzuki K Grigorova I Phan TG Kelly LM Cyster JG. Visualizing B cell capture of cognate antigen from follicular dendritic cells. J Exp Med (2009) 206:1485–93. 10.1084/jem.20090209 19506051 PMC2715076

[53] Avalos AM Ploegh HL. Early BCR events and antigen capture, processing, and loading on MHC class II on B cells. Front Immunol (2014) 5:92. 10.3389/fimmu.2014.00092 24653721 PMC3948085

[54] Roche PA Furuta K. The ins and outs of MHC class II-mediated antigen processing and presentation. Nat Rev Immunol (2015) 15:203–16. 10.1038/nri3818 25720354 PMC6314495

[55] Cocucci E Aguet F Boulant S Kirchhausen T. The first five seconds in the life of a clathrin-coated pit. Cell (2012) 150:495–507. 10.1016/j.cell.2012.05.047 22863004 PMC3413093

[56] Busman-Sahay K Drake L Sitaram A Marks M Drake JR. Cis and trans regulatory mechanisms control AP2-mediated B cell receptor endocytosis via select tyrosine-based motifs. PLoS One (2013) 8:e54938. 10.1371/journal.pone.0054938 23372794 PMC3553015

[57] Boulant S Kural C Zeeh JC Ubelmann F Kirchhausen T. Actin dynamics counteract membrane tension during clathrin-mediated endocytosis. Nat Cell Biol (2011) 13:1124–31. 10.1038/ncb2307 21841790 PMC3167020

[58] Granger E Mcnee G Allan V Woodman P. The role of the cytoskeleton and molecular motors in endosomal dynamics. Semin Cell Dev Biol (2014) 31:20–9. 10.1016/j.semcdb.2014.04.011 24727350 PMC4071412

[59] Katkere B Rosa S Drake JR. The Syk-binding ubiquitin ligase c-Cbl mediates signaling-dependent B cell receptor ubiquitination and B cell receptor-mediated antigen processing and presentation. J Biol Chem (2012) 287:16636–44. 10.1074/jbc.M112.357640 22451666 PMC3351345

[60] Satpathy S Wagner SA Beli P Gupta R Kristiansen TA Malinova D Systems-wide analysis of BCR signalosomes and downstream phosphorylation and ubiquitylation. Mol Syst Biol (2015) 11:810. 10.15252/msb.20145880 26038114 PMC4501846

[61] Song W Cho H Cheng P Pierce SK. Entry of B cell antigen receptor and antigen into class II peptide-loading compartment is independent of receptor cross-linking. J Immunol (1995) 155:4255–63. 7594583

[62] Stoddart A Jackson AP Brodsky FM. Plasticity of B cell receptor internalization upon conditional depletion of clathrin. Mol Biol Cell (2005) 16:2339–48. 10.1091/mbc.E05-01-0025 15716350 PMC1087239

[63] Davis RE Ngo VN Lenz G Tolar P Young RM Romesser PB Chronic active B-cell-receptor signalling in diffuse large B-cell lymphoma. Nature (2010) 463:88–92. 10.1038/nature08638 20054396 PMC2845535

[64] Malhotra S Kovats S Zhang W Coggeshall KM. Vav and Rac activation in B cell antigen receptor endocytosis involves Vav recruitment to the adapter protein LAB. J Biol Chem (2009) 284:36202–12. 10.1074/jbc.M109.040089 19858206 PMC2794736

[65] Clark MR Tanaka A Powers SE Veselits M. Receptors, subcellular compartments and the regulation of peripheral B cell responses: the illuminating state of anergy. Mol Immunol (2011) 48:1281–6. 10.1016/j.molimm.2010.10.024 21144589 PMC3089810

[66] Chaturvedi A Martz R Dorward D Waisberg M Pierce SK. Endocytosed BCRs sequentially regulate MAPK and Akt signaling pathways from intracellular compartments. Nat Immunol (2011) 12:1119–26. 10.1038/ni.2116 21964606 PMC3746798

[67] Cloutier M Gauthier C Fortin JS Thibodeau J. The invariant chain p35 isoform promotes formation of nonameric complexes with MHC II molecules. Immunol Cell Biol (2014) 92:553–6. 10.1038/icb.2014.17 24638068

[68] Cresswell P Roche PA Invariant chain-MHC class II complexes: always odd and never invariant. Immunol Cell Biol (2014) 92:471–2. 10.1038/icb.2014.36 24777311 PMC6322397

[69] Dugast M Toussaint H Dousset C Benaroch P. AP2 clathrin adaptor complex, but not AP1, controls the access of the major histocompatibility complex (MHC) class II to endosomes. J Biol Chem (2005) 280:19656–64. 10.1074/jbc.M501357200 15749704

[70] McCormick PJ Martina JA Bonifacino JS. Involvement of clathrin and AP-2 in the trafficking of MHC class II molecules to antigen-processing compartments. Proc Natl Acad Sci U S A (2005) 102:7910–5. 10.1073/pnas.0502206102 15911768 PMC1138261

[71] Neefjes J. CIIV, MIIC and other compartments for MHC class II loading. Eur J Immunol (1999) 29:1421–5. 10.1002/(SICI)1521-4141(199905)29:05<1421::AID-IMMU1421>3.0.CO;2-C 10359095

[72] Rovere P Zimmermann VS Forquet F Demandolx D Trucy J Ricciardi-Castagnoli P Dendritic cell maturation and antigen presentation in the absence of invariant chain. Proc Natl Acad Sci U S A (1998) 95:1067–72. 10.1073/pnas.95.3.1067 9448286 PMC18674

[73] Zimmermann VS Rovere P Trucy J Serre K Machy P Forquet F Engagement of B cell receptor regulates the invariant chain-dependent MHC class II presentation pathway. J Immunol (1999) 162:2495–502. 10072488

[74] Viville S Neefjes J Lotteau V Dierich A Lemeur M Ploegh H Mice lacking the MHC class II-associated invariant chain. Cell (1993) 72:635–48. 10.1016/0092-8674(93)90081-Z 7679955

[75] Brachet V Raposo G Amigorena S Mellman I. Ii chain controls the transport of major histocompatibility complex class II molecules to and from lysosomes. J Cell Biol (1997) 137:51–65. 10.1083/jcb.137.1.51 9105036 PMC2139866

[76] Lankar D Vincent-Schneider H Briken V Yokozeki T Raposo G Bonnerot C. Dynamics of major histocompatibility complex class II compartments during B cell receptor-mediated cell activation. J Exp Med (2002) 195:461–72. 10.1084/jem.20011543 11854359 PMC2193618

[77] Zhang M Veselits M O’neill S Hou P Reddi AL Berlin I Ubiquitinylation of Ig beta dictates the endocytic fate of the B cell antigen receptor. J Immunol (2007) 179:4435–43. 10.4049/jimmunol.179.7.4435 17878339

[78] Barroso M Tucker H Drake L Nichol K Drake JR Antigen-B cell receptor complexes associate with intracellular major histocompatibility complex (MHC) class II molecules. J Biol Chem (2015) 290:27101–12. 10.1074/jbc.M115.649582 26400081 PMC4646406

[79] Busman-Sahay K Sargent E Harton JA Drake JR. The Ia.2 epitope defines a subset of lipid raft-resident MHC class II molecules crucial to effective antigen presentation. J Immunol (2011) 186:6710–7. 10.4049/jimmunol.1100336 21543648 PMC4860613

[80] Dixon AM Drake L Hughes KT Sargent E Hunt D Harton JA Differential transmembrane domain GXXXG motif pairing impacts major histocompatibility complex (MHC) class II structure. J Biol Chem (2014) 289:11695–703. 10.1074/jbc.M113.516997 24619409 PMC4002079

[81] Blum JS Wearsch PA Cresswell P. Pathways of antigen processing. Annu Rev Immunol (2013) 31:443–73. 10.1146/annurev-immunol-032712-095910 23298205 PMC4026165

[82] van Kasteren SI Overkleeft HS. Endo-lysosomal proteases in antigen presentation. Curr Opin Chem Biol (2014) 23:8–15. 10.1016/j.cbpa.2014.08.011 25213682

[83] West LC Cresswell P. Expanding roles for GILT in immunity. Curr Opin Immunol (2013) 25:103–8. 10.1016/j.coi.2012.11.006 23246037 PMC4287230

[84] Honey K Rudensky AY. Lysosomal cysteine proteases regulate antigen presentation. Nat Rev Immunol (2003) 3:472–82. 10.1038/nri1110 12776207

[85] Driessen C Lennon-Dumenil AM Ploegh HL. Individual cathepsins degrade immune complexes internalized by antigen-presenting cells via Fcgamma receptors. Eur J Immunol (2001) 31:1592–601. 10.1002/1521-4141(200105)31:5<1592::AID-IMMU1592>3.0.CO;2-K 11465117

[86] Shi GP Bryant RA Riese R Verhelst S Driessen C Li Z Role for cathepsin F in invariant chain processing and major histocompatibility complex class II peptide loading by macrophages. J Exp Med (2000) 191:1177–86. 10.1084/jem.191.7.1177 10748235 PMC2193169

[87] Vasiljeva O Dolinar M Turk V Turk B. Recombinant human cathepsin H lacking the mini chain is an endopeptidase. Biochemistry (2003) 42:13522–8. 10.1021/bi035355k 14621998

[88] Hsing LC Rudensky AY. The lysosomal cysteine proteases in MHC class II antigen presentation. Immunol Rev (2005) 207:229–41. 10.1111/j.0105-2896.2005.00310.x 16181340

[89] Tolosa E Li W Yasuda Y Wienhold W Denzin LK Lautwein A Cathepsin V is involved in the degradation of invariant chain in human thymus and is overexpressed in myasthenia gravis. J Clin Invest (2003) 112:517–26. 10.1172/JCI18028 12925692 PMC171390

[90] Moss CX Villadangos JA Watts C. Destructive potential of the aspartyl protease cathepsin D in MHC class II-restricted antigen processing. Eur J Immunol (2005) 35:3442–51. 10.1002/eji.200535320 16259009

[91] Burster T Reich M Zaidi N Voelter W Boehm BO Kalbacher H. Cathepsin E regulates the presentation of tetanus toxin C-fragment in PMA activated primary human B cells. Biochem Biophys Res Commun (2008) 377:1299–303. 10.1016/j.bbrc.2008.10.162 18996084

[92] Reich M Spindler KD Burret M Kalbacher H Boehm BO Burster T. Cathepsin A is expressed in primary human antigen-presenting cells. Immunol Lett (2010) 128:143–7. 10.1016/j.imlet.2009.11.010 19954752

[93] Burster T Macmillan H Hou T Boehm BO Mellins ED. Cathepsin G: roles in antigen presentation and beyond. Mol Immunol (2010) 47:658–65. 10.1016/j.molimm.2009.10.003 19910052 PMC4159238

[94] Manoury B Mazzeo D Li DN Billson J Loak K Benaroch P Asparagine endopeptidase can initiate the removal of the MHC class II invariant chain chaperone. Immunity (2003) 18:489–98. 10.1016/S1074-7613(03)00085-2 12705852

[95] Manoury B Hewitt EW Morrice N Dando PM Barrett AJ Watts C. An asparaginyl endopeptidase processes a microbial antigen for class II MHC presentation. Nature (1998) 396:695–9. 10.1038/25379 9872320

[96] Beck H Schwarz G Schroter CJ Deeg M Baier D Stevanovic S Cathepsin S and an asparagine-specific endoprotease dominate the proteolytic processing of human myelin basic protein in vitro. Eur J Immunol (2001) 31:3726–36. 10.1002/1521-4141(200112)31:12<3726::AID-IMMU3726>3.0.CO;2-O 11745393

[97] Manoury B Mazzeo D Fugger L Viner N Ponsford M Streeter H Destructive processing by asparagine endopeptidase limits presentation of a dominant T cell epitope in MBP. Nat Immunol (2002) 3:169–74. 10.1038/ni754 11812994

[98] Matthews SP Werber I Deussing J Peters C Reinheckel T Watts C. Distinct protease requirements for antigen presentation in vitro and in vivo. J Immunol (2010) 184:2423–31. 10.4049/jimmunol.0901486 20164435

[99] Shirahama-Noda K Yamamoto A Sugihara K Hashimoto N Asano M Nishimura M Biosynthetic processing of cathepsins and lysosomal degradation are abolished in asparaginyl endopeptidase-deficient mice. J Biol Chem (2003) 278:33194–9. 10.1074/jbc.M302742200 12775715

[100] Shi GP Villadangos JA Dranoff G Small C Gu L Haley KJ Cathepsin S required for normal MHC class II peptide loading and germinal center development. Immunity (1999) 10:197–206. 10.1016/S1074-7613(00)80020-5 10072072

[101] Davidson HW West MA Watts C. Endocytosis, intracellular trafficking, and processing of membrane IgG and monovalent antigen/membrane IgG complexes in B lymphocytes. J Immunol (1990) 144:4101–9. 2187925

[102] Cheng PC Steele CR Gu L Song W Pierce SK. MHC class II antigen processing in B cells: accelerated intracellular targeting of antigens. J Immunol (1999) 162:7171–80. 10358163

[103] Burster T Beck A Tolosa E Marin-Esteban V Rotzschke O Falk K Cathepsin G, and not the asparagine-specific endoprotease, controls the processing of myelin basic protein in lysosomes from human B lymphocytes. J Immunol (2004) 172:5495–503. 10.4049/jimmunol.172.9.5495 15100291

[104] Sealy L Mota F Rayment N Tatnell P Kay J Chain B. Regulation of cathepsin E expression during human B cell differentiation in vitro. Eur J Immunol (1996) 26:1838–43. 10.1002/eji.1830260826 8765029

[105] Phipps-Yonas H Semik V Hastings KT. GILT expression in B cells diminishes cathepsin S steady-state protein expression and activity. Eur J Immunol (2013) 43:65–74. 10.1002/eji.201242379 23012103 PMC3706190

[106] Delamarre L Pack M Chang H Mellman I Trombetta ES. Differential lysosomal proteolysis in antigen-presenting cells determines antigen fate. Science (2005) 307:1630–4. 10.1126/science.1108003 15761154

[107] Kim A Hartman IZ Poore B Boronina T Cole RN Song N Divergent paths for the selection of immunodominant epitopes from distinct antigenic sources. Nat Commun (2014) 5:5369. 10.1038/ncomms6369 25413013 PMC4241505

[108] Sadegh-Nasseri S Kim A. Exogenous antigens bind MHC class II first, and are processed by cathepsins later. Mol Immunol (2015) 68:81–4. 10.1016/j.molimm.2015.07.018 26254987 PMC4623955

[109] Brooks K Knight AM. Lowering the affinity between antigen and the B cell receptor can enhance antigen presentation. Eur J Immunol (2004) 34:837–43. 10.1002/eji.200324357 14991613

[110] Devanaboyina SC Lynch SM Ober RJ Ram S Kim D Puig-Canto A The effect of pH dependence of antibody-antigen interactions on subcellular trafficking dynamics. MAbs (2013) 5:851–9. 10.4161/mabs.26389 24492341 PMC3896599

[111] Simitsek PD Campbell DG Lanzavecchia A Fairweather N Watts C. Modulation of antigen processing by bound antibodies can boost or suppress class II major histocompatibility complex presentation of different T cell determinants. J Exp Med (1995) 181:1957–63. 10.1084/jem.181.6.1957 7539034 PMC2192058

[112] Gondre-Lewis TA Moquin AE Drake JR. Prolonged antigen persistence within nonterminal late endocytic compartments of antigen-specific B lymphocytes. J Immunol (2001) 166:6657–64. 10.4049/jimmunol.166.11.6657 11359820

[113] Guce AI Mortimer SE Yoon T Painter CA Jiang W Mellins ED HLA-DO acts as a substrate mimic to inhibit HLA-DM by a competitive mechanism. Nat Struct Mol Biol (2013) 20:90–8. 10.1038/nsmb.2460 23222639 PMC3537886

[114] Busch R Rinderknecht CH Roh S Lee AW Harding JJ Burster T Achieving stability through editing and chaperoning: regulation of MHC class II peptide binding and expression. Immunol Rev (2005) 207:242–60. 10.1111/j.0105-2896.2005.00306.x 16181341

[115] Schulze MS Wucherpfennig KW. The mechanism of HLA-DM induced peptide exchange in the MHC class II antigen presentation pathway. Curr Opin Immunol (2012) 24:105–11. 10.1016/j.coi.2011.11.004 22138314 PMC3288754

[116] Mellins E Smith L Arp B Cotner T Celis E Pious D. Defective processing and presentation of exogenous antigens in mutants with normal HLA class II genes. Nature (1990) 343:71–4. 10.1038/343071a0 1967485

[117] Martin WD Hicks GG Mendiratta SK Leva HI Ruley HE Van Kaer L. H2-M mutant mice are defective in the peptide loading of class II molecules, antigen presentation, and T cell repertoire selection. Cell (1996) 84:543–50. 10.1016/S0092-8674(00)81030-2 8598041

[118] Denzin LK Hammond C Cresswell P. HLA-DM interactions with intermediates in HLA-DR maturation and a role for HLA-DM in stabilizing empty HLA-DR molecules. J Exp Med (1996) 184:2153–65. 10.1084/jem.184.6.2153 8976171 PMC2196380

[119] Pashine A Busch R Belmares MP Munning JN Doebele RC Buckingham M Interaction of HLA-DR with an acidic face of HLA-DM disrupts sequence-dependent interactions with peptides. Immunity (2003) 19:183–92. 10.1016/S1074-7613(03)00200-0 12932352

[120] Sherman MA Weber DA Jensen PE. DM enhances peptide binding to class II MHC by release of invariant chain-derived peptide. Immunity (1995) 3:197–205. 10.1016/1074-7613(95)90089-6 7648393

[121] Sloan VS Cameron P Porter G Gammon M Amaya M Mellins E Mediation by HLA-DM of dissociation of peptides from HLA-DR. Nature (1995) 375:802–6. 10.1038/375802a0 7596415

[122] Hou T Macmillan H Chen Z Keech CL Jin X Sidney J An insertion mutant in DQA1*0501 restores susceptibility to HLA-DM: implications for disease associations. J Immunol (2011) 187:2442–52. 10.4049/jimmunol.1100255 21775680 PMC3159820

[123] Yin L Maben ZJ Becerra A Stern LJ. Evaluating the role of HLA-DM in MHC class II-peptide association reactions. J Immunol (2015) 195:706–16. 10.4049/jimmunol.1403190 26062997 PMC4490944

[124] Kropshofer H Arndt SO Moldenhauer G Hammerling GJ Vogt AB. HLA-DM acts as a molecular chaperone and rescues empty HLA-DR molecules at lysosomal pH. Immunity (1997) 6:293–302. 10.1016/S1074-7613(00)80332-5 9075930

[125] Jiang W Strohman MJ Somasundaram S Ayyangar S Hou T Wang N pH-susceptibility of HLA-DO tunes DO/DM ratios to regulate HLA-DM catalytic activity. Sci Rep (2015) 5:17333. 10.1038/srep17333 26610428 PMC4661524

[126] Chen X Laur O Kambayashi T Li S Bray RA Weber DA Regulated expression of human histocompatibility leukocyte antigen (HLA)-DO during antigen-dependent and antigen-independent phases of B cell development. J Exp Med (2002) 195:1053–62. 10.1084/jem.20012066 11956296 PMC2193689

[127] Glazier KS Hake SB Tobin HM Chadburn A Schattner EJ Denzin LK. Germinal center B cells regulate their capability to present antigen by modulation of HLA-DO. J Exp Med (2002) 195:1063–9. 10.1084/jem.20012059 11956297 PMC2193692

[128] Draghi NA Denzin LK. H2-O, a MHC class II-like protein, sets a threshold for B-cell entry into germinal centers. Proc Natl Acad Sci U S A (2010) 107:16607–12. 10.1073/pnas.1004664107 20807742 PMC2944729

[129] Mellins ED Stern LJ. HLA-DM and HLA-DO, key regulators of MHC-II processing and presentation. Curr Opin Immunol (2014) 26:115–22. 10.1016/j.coi.2013.11.005 24463216 PMC3944065

[130] Gu Y Jensen PE Chen X. Immunodeficiency and autoimmunity in H2-O-deficient mice. J Immunol (2013) 190:126–37. 10.4049/jimmunol.1200993 23209323

[131] Pos W Sethi DK Call MJ Schulze MS Anders AK Pyrdol J Crystal structure of the HLA-DM-HLA-DR1 complex defines mechanisms for rapid peptide selection. Cell (2012) 151:1557–68. 10.1016/j.cell.2012.11.025 23260142 PMC3530167

[132] Fallas JL Yi W Draghi NA O’rourke HM Denzin LK. Expression patterns of H2-O in mouse B cells and dendritic cells correlate with cell function. J Immunol (2007) 178:1488–97. 10.4049/jimmunol.178.3.1488 17237397

[133] Yoon T Macmillan H Mortimer SE Jiang W Rinderknecht CH Stern LJ Mapping the HLA-DO/HLA-DM complex by FRET and mutagenesis. Proc Natl Acad Sci U S A (2012) 109:11276–81. 10.1073/pnas.1113966109 22733780 PMC3396517

[134] Liljedahl M Kuwana T Fung-Leung WP Jackson MR Peterson PA Karlsson L. HLA-DO is a lysosomal resident which requires association with HLA-DM for efficient intracellular transport. EMBO J (1996) 15:4817–24. 8890155 PMC452218

[135] Denzin LK. Inhibition of HLA-DM mediated MHC class II peptide loading by HLA-DO promotes self tolerance. Front Immunol (2013) 4:465. 10.3389/fimmu.2013.00465 24381574 PMC3865790

[136] Poluektov YO Kim A Sadegh-Nasseri S HLA-DO and its role in MHC class II antigen presentation. Front Immunol (2013) 4:260 10.3389/fimmu.2013.00260 24009612 PMC3756479

[137] Chen X Jensen PE. Biological function of HLA-DO (H2-O). Crit Rev Immunol (2014) 34:215–25. 10.1615/CritRevImmunol.2014009999 24941074

[138] Poluektov YO Kim A Hartman IZ Sadegh-Nasseri S HLA-DO as the optimizer of epitope selection for MHC class II antigen presentation. PLoS One (2013) 8:e71228 10.1371/journal.pone.0071228 23951115 PMC3738515

[139] Santambrogio L Sato AK Carven GJ Belyanskaya SL Strominger JL Stern LJ Extracellular antigen processing and presentation by immature dendritic cells. Proc Natl Acad Sci U S A (1999) 96:15056–61. 10.1073/pnas.96.26.15056 10611337 PMC24772

[140] Xiu F Cote MH Bourgeois-Daigneault MC Brunet A Gauvreau ME Shaw A Cutting edge: HLA-DO impairs the incorporation of HLA-DM into exosomes. J Immunol (2011) 187:1547–51. 10.4049/jimmunol.1100199 21768396

[141] van Lith M van Ham M Griekspoor A Tjin E Verwoerd D Calafat J Regulation of MHC class II antigen presentation by sorting of recycling HLA-DM/DO and class II within the multivesicular body. J Immunol (2001) 167:884–92. 10.4049/jimmunol.167.2.884 11441095

[142] Jahnke M Trowsdale J Kelly AP. Ubiquitination of HLA-DO by MARCH family E3 ligases. Eur J Immunol (2013) 43:1153–61. 10.1002/eji.201243043 23400868 PMC3655539

[143] Fallas JL Tobin HM Lou O Guo D Sant’angelo DB Denzin LK. Ectopic expression of HLA-DO in mouse dendritic cells diminishes MHC class II antigen presentation. J Immunol (2004) 173:1549–60. 10.4049/jimmunol.173.3.1549 15265882

[144] Macmillan H Strohman MJ Ayyangar S Jiang W Rajasekaran N Spura A The MHC class II cofactor HLA-DM interacts with Ig in B cells. J Immunol (2014) 193:2641–50. 10.4049/jimmunol.1400075 25098292 PMC4157100

[145] Cho KJ Walseng E Ishido S Roche PA. Ubiquitination by March-I prevents MHC class II recycling and promotes MHC class II turnover in antigen-pre senting cells. Proc Natl Acad Sci U S A (2015) 112:10449–54. 10.1073/pnas.1507981112 26240324 PMC4547296

[146] Alt FW Yancopoulos GD Blackwell TK Wood C Thomas E Boss M Ordered rearrangement of immunoglobulin heavy chain variable region segments. EMBO J (1984) 3:1209–19. 6086308 10.1002/j.1460-2075.1984.tb01955.x PMC557501

[147] Pillai S Baltimore D. Myristoylation and the post-translational acquisition of hydrophobicity by the membrane immunoglobulin heavy-chain polypeptide in B lymphocytes. Proc Natl Acad Sci U S A (1987) 84:7654–8. 10.1073/pnas.84.21.7654 3118373 PMC299358

[148] Karasuyama H Rolink A Shinkai Y Young F Alt FW Melchers F. The expression of Vpre-B/lambda 5 surrogate light chain in early bone marrow precursor B cells of normal and B cell-deficient mutant mice. Cell (1994) 77:133–43. 10.1016/0092-8674(94)90241-0 8156589

[149] Lombard-Platet S Fisher AG Meyer V Ceredig R. Expression of functional MHC class II molecules by a mouse pro-B cell clone. Dev Immunol (1995) 4:85–92. 10.1155/1995/10359 9700358 PMC2275953

[150] Matsuki Y Ohmura-Hoshino M Goto E Aoki M Mito-Yoshida M Uematsu M Novel regulation of MHC class II function in B cells. EMBO J (2007) 26:846–54. 10.1038/sj.emboj.7601556 17255932 PMC1794403

[151] Matza D Lantner F Bogoch Y Flaishon L Hershkoviz R Shachar I. Invariant chain induces B cell maturation in a process that is independent of its chaperonic activity. Proc Natl Acad Sci U S A (2002) 99:3018–23. 10.1073/pnas.052703299 11867743 PMC122465

[152] Matza D Kerem A Shachar I Invariant chain, a chain of command. Trends Immunol (2003) 24:264–8. 10.1016/S1471-4906(03)00073-5 12738421

[153] Schneppenheim J Huttl S Kruchen A Fluhrer R Muller I Saftig P Signal-peptide-peptidase-like 2a is required for CD74 intramembrane proteolysis in human B cells. Biochem Biophys Res Commun (2014) 451:48–53. 10.1016/j.bbrc.2014.07.051 25035924 PMC4476656

[154] Schroder B The multifaceted roles of the invariant chain CD74 – more than just a chaperone. Biochim Biophys Acta (2016) 1863:1269–81. 10.1016/j.bbamcr.2016.03.026 27033518

[155] Rolink AG Brocker T Bluethmann H Kosco-Vilbois MH Andersson J Melchers F. Mutations affecting either generation or survival of cells influence the pool size of mature B cells. Immunity (1999) 10:619–28. 10.1016/S1074-7613(00)80061-8 10367907

[156] Chen X Jensen PE. MHC class II antigen presentation and immunological abnormalities due to deficiency of MHC class II and its associated genes. Exp Mol Pathol (2008) 85:40–4. 10.1016/j.yexmp.2008.03.011 18547561 PMC2568888

[157] Reith W Mach B. The bare lymphocyte syndrome and the regulation of MHC expression. Annu Rev Immunol (2001) 19:331–73. 10.1146/annurev.immunol.19.1.331 11244040

[158] Lu TT Cyster JG. Integrin-mediated long-term B cell retention in the splenic marginal zone. Science (2002) 297:409–12. 10.1126/science.1071632 12130787

[159] Pillai S Cariappa A Moran ST. Marginal zone B cells. Annu Rev Immunol (2005) 23:161–96. 10.1146/annurev.immunol.23.021704.115728 15771569

[160] Chen X Jensen PE. The expression of HLA-DO (H2-O) in B lymphocytes. Immunol Res (2004) 29:19–28. 10.1385/IR:29:1-3:019 15181267

[161] Victora GD Nussenzweig MC. Germinal centers. Annu Rev Immunol (2012) 30:429–57. 10.1146/annurev-immunol-020711-075032 22224772

[162] Klein U Dalla-Favera R. Germinal centres: role in B-cell physiology and malignancy. Nat Rev Immunol (2008) 8:22–33. 10.1038/nri2217 18097447

[163] Pavri R Nussenzweig MC. AID targeting in antibody diversity. Adv Immunol (2011) 110:1–26. 10.1016/B978-0-12-387663-8.00005-3 21762814

[164] Bannard O Mcgowan SJ Ersching J Ishido S Victora GD Shin JS Ubiquitin-mediated fluctuations in MHC class II facilitate efficient germinal center B cell responses. J Exp Med (2016) 213:993–1009. 10.1084/jem.20151682 27162138 PMC4886361

[165] Davidson HW Reid PA Lanzavecchia A Watts C. Processed antigen binds to newly synthesized MHC class II molecules in antigen-specific B lymphocytes. Cell (1991) 67:105–16. 10.1016/0092-8674(91)90575-J 1913812

[166] Thaunat O Granja AG Barral P Filby A Montaner B Collinson L Asymmetric segregation of polarized antigen on B cell division shapes presentation capacity. Science (2012) 335:475–9. 10.1126/science.1214100 22282815

[167] Sartoris S Tosi G De Lerma Barbaro A Cestari T Accolla RS. Active suppression of the class II transactivator-encoding AIR-1 locus is responsible for the lack of major histocompatibility complex class II gene expression observed during differentiation from B cells to plasma cells. Eur J Immunol (1996) 26:2456–60. 10.1002/eji.1830261028 8898960

[168] Shimoda M Li T Pihkala JP Koni PA. Role of MHC class II on memory B cells in post-germinal center B cell homeostasis and memory response. J Immunol (2006) 176:2122–33. 10.4049/jimmunol.176.4.2122 16455968

